# The prevalence of hoarseness among health care professionals: time trends and effect of remediation in working conditions in 2007–2018

**DOI:** 10.1007/s00420-022-01934-9

**Published:** 2022-11-04

**Authors:** Liisa Vilén, Janne Atosuo, Tuula Putus

**Affiliations:** 1 Department of Clinical Medicine, Environmental Medicine and Occupational Health, University of Turku, 20014 Turku, Finland; 2 Clinical Research Unit TROSSI, University of Turku, Turku, Finland; 3 Department of Biotechnology, The Laboratory of Immunochemistry, University of Turku, Turku, Finland

**Keywords:** Hoarseness, Indoor environment quality, Health care professionals, Longitudinal study, Hospital, Primary health care

## Abstract

**Purpose:**

The purpose of this study was to investigate time trends in the prevalence of hoarseness among health care professionals in primary health care units (PHC) and in hospitals from 2007 to 2018. Moreover, purpose was to discover potential indoor environmental quality (IEQ) risk factors as well as to determine the effect of the remediation of the indoor air problems on the prevalence of hoarseness.

**Methods:**

The health status was collected from all employees in these units/hospitals (*N* = 1564/1199) with questionnaires and the follow-ups were carried out as an open cohort. Based on building condition inspections, buildings were classified to be an “exposed” or “reference” buildings by third-party experts. The before and after remediation results were compared to reference buildings.

**Results:**

During follow-up, hoarseness has not increased in those PHC units with good IEQ. In the pilot study, the prevalence of hoarseness in non-exposed reference building was 5.9%, and it stayed approximately at the same level throughout the follow-up. Whereas in buildings with an IEQ problem the prevalence of hoarseness varied between 16.2 and 36.1% and it decreased to 11.4% after the remediations. In a large hospital with severe IEQ problems, the prevalence of hoarseness was 39.1%, and in hospital buildings with a milder exposure 23.3%. The most important risk factors for hoarseness were asthma, allergic rhinitis and IEQ problems.

**Conclusion:**

A good indoor environment and the remediation of damaged buildings seem to promote a better condition of the voice in health care workers.

## Introduction

A healthy and well-functional voice is a valuable tool in many occupations. Verbal communication is increasingly necessary, and the voice is subject to many stressors, such as noise, dusts, too high and too low temperatures, man-made mineral fibers, allergens, and chemicals. Due to the Cov-19-pandemic, the use of face masks has increased especially in health care, thus there is a lack of facial expressions and communication with patients is disturbed by such protective equipment. In addition, the obligatory distance between the patient and the staff makes a clear and strong voice even more important than before. It can therefore be said that the voice plays an important role in achieving care satisfaction and patient safety.

Hoarseness is a common symptom in adults, mainly caused by the prolonged use of the voice, smoking or other exposure to laryngeal irritants, prolonged airway inflammation or allergies, as well as long-term treatment of the airways with inhaled corticosteroids, e.g. for asthma (House and Fisher 2017). In addition to prolonged voice use, health care personnel in primary health care (PHC) units and hospitals have considerable exposure to agents in their work surroundings, such as various cleaning chemicals, surgical fumes, anesthesia gases, and medical drugs, which are also known to cause respiratory symptoms (El-Helaly et al. 2016; Polovich and Gieseker 2011; Stoeva 2021). Despite all this, not much attention has been paid to the prevalence of hoarseness among healthcare professionals, but research has focused on typical speech professions, such as teachers (Byeon 2017, 2019; Morawska and Niebudek-Bogusz 2017; Rantala et al. 2012; Roy et al. 2005; Vertanen-Greis et al. 2018, 2020).

Based on earlier studies among health care professional, the prevalence of hoarseness among nurses has tripled over the past twenty years in Finland, which is a worrying change (Sala et al. 2001; Vilén et al. 2022; Vilén and Putus 2021).

Therefore, in this longitudinal study, our aims were to investigate the possible changes in prevalence of hoarseness among health care professionals over the period 2007–2018 and discover potential indoor environmental quality (IEQ) risk factors for hoarseness among health care professionals working in different units. In addition, in those buildings where remediation has been carried out due to indoor air problems, the effect of remediation on the health status was investigated before and after the repairs. Our hypothesis was that a thorough remediation of buildings promotes the health of employees and reduces symptoms.

## Material and methods

### Participating units and schedule of monitoring

The study commenced in 2007 with a pilot study (*N*_p(=pilot)_ = 55) in two wards for the elderly, one with an indoor air problem (*n*_p_ = 37) and the other serving as a reference ward (*n*_p_ = 18). In the second phase (2008–2018), 16 PHC units were examined (*N*_2_ = 1128), 12 damaged buildings (*n*_2_ = 856) and four reference buildings (*n*_2_ = 272). The response rates varied from 68 to 90%.

Five years after the pilot study in 2011, the third phase was launched using two hospital buildings (*N*_3_ = 1199) in the same city: one building with severe moisture-damages (*n*_3_ = 973) and one with minor damages as a reference building (*n*_3_ = 226). The response rate in the problem building was 97.2% and in the reference building 55.6%.

In the fourth phase of the study (2010–2016), as four of the damaged PHC buildings in Phase 2 had been remediated, a follow-up survey was sent to all employees in the repaired units and also to four reference buildings with no known IEQ problem in the same region. Three of the reference units that had participated in Phase 2 survey also participated in the follow-up, and one reference PHC unit participated twice. At the beginning of Phase 4, there were 275 exposed and 272 reference participants. These two groups were compared to each other before the renovations.

After the repairs, the results from 328 participants working in all the renovated buildings were compared to 102 non-exposed participants from the follow-up survey: the follow-up survey was conducted the same year as the renovation of the problem buildings was completed. In Phase 4, the interval between surveys before and after the repairs was 2–3 years in different PHC units (Table 1).

**Table 1:** Time schedule of the study and number of units

	2007	2008	2009	2010	2011	2012	2013	2014	2015	2016	2017	2018
Pilot	2											
Phase 2 PHC units		4	4	4	1		1		1			1
Phase 3 hospitals					2							
Phase 4 repair and follow-up (case + ref.pair)				1 + 1		1 + 1		1 + 1		1 + 1		

### Data collection

The data was collected from all employees in these units/hospitals, initially by sending mailed questionnaires, but as from the beginning of Phase 2 via e-mail. The follow-ups were carried out as an open cohort, meaning that new employees recruited during the follow-up could participate the surveys and employees who had retired or moved to other regions were lost from the follow-up. Nonetheless, a total of 336 individuals responded at least twice during the follow-up.

The questionnaire used was validated in 2005 and has been in regular use ever since (Savilahti et al. 2005). The questionnaire is comprised of 53 questions, containing questions on the work environment, different exposures, the duration of exposure, subjective indoor environment quality (IEQ) factors, respiratory and general symptoms, occurrence of respiratory infections, use of health care services, medication and sick leave and lifestyle factors, such as smoking and pet owning. In the pilot study, we had no questions about work-related stress or work overload, these were added after the piloting. All the surveys between 2008 and 2018 had similar questions about work-related stress or work overload.

### Exposure data

All the evaluations have been done according to national guidelines, which are based on the Health Protection Act (Husman 1999) and its Decree of the Ministry of Social Affairs and Health on Health-related Conditions of Housing and Other Residential Buildings and Qualification Requirements for Third-party Experts (545/2015). All buildings were inspected by qualified construction experts and microbiological samples were taken from surfaces and materials and moisture damage indicator microbes were cultivated and identified according to guidelines. The classification of buildings as an “exposed building” or “reference building’ was done by external experts. Exposure assessment data was obtained from the employer.

### Data analysis

In the analysis of the health data, the symptom data was coded into dichotomous variables; the prevalence of symptoms reported ‘daily’ or ‘every week’ were combined to be 'symptomatic´, and alternatives ‘more seldom’ or ‘never’ were combined to be 'no symptoms´. The same kind of combining was done with the reported IEQ factors. Smoking was defined as at least one cigarette per day for at least one year. Those who had stopped smoking for less than 6 months were classified as smokers. Information on asthma, allergic rhinitis, and atopic eczema was requested under doctor diagnosed diseases.

Statistical analyses were made with SPSS Statistics 26–version (IBM Corp. Armonk, NY). In the statistical analysis, a *p* value below 0.05 was considered statistically significant. For odds ratios, a confidence interval of 95% was calculated. Logistic regression models were used when controlling for confounding variables, such as age, gender, and smoking.

In the statistical analysis of the follow-up, dichotomous variables between the cluster samples (non-paired data of exposure group and reference group) were compared to a Chi-Square (*Χ*^2^) test. After the follow-up survey, the results prior to remediation were compared to the respective results in the same unit after the repair. The paired data of the same respondent pre- and post-renovation were compared to McNemar-Bowker’s test. Finally, a logistic regression model was used to control for confounding variables to determine the effect of the remediation.

## Results

### Pilot study and Phase 2

The prevalence of hoarseness in the pilot study was 5.9% among the non-exposed group and 36% in the exposure group. During the first four years of Phase 2, the prevalence remained almost the same in the non-exposure group (5.6%) and in the exposure groups somewhat lower (21.2%) than in the pilot study. The prevalence of hoarseness among the IEQ problem group was significantly higher than in the reference group in the Phase 2 study population. Descriptive data of the pilot study (2007) and PHC units (2008–2011) are presented in Table 2.

**Table 2:** Description of the pilot study (2007) and primary health care units’ years 2008–11 in Phase 2

	Pilot study, exp	Pilot study, ref	Pilot study, total	PHC units, exp	PHC units, ref	PHC units total	*p* value
No. of buildings	1	1	2	12	4	16	
Mean age (y)	41.4	43.3	42.0	45.1	43.5	44.9	
Men women Total	3 (8.1) 34 (91.9) 37	0 18 (100) 18	3 (6) 52 (94) 55	52 (6.1) 804 (93.9) 856	16 (5.9) 256 (94.1) 272	68 (6.0) 1060 (94.0) 1128	n.s.
Smokers	10 (29.4)	4 (23.5)	14 (27.5)	102 (14.2)	26 (10.5)	128 (13.3)	0.01
passive Smoking	3 (9.1)	1 (6.7)	3 (5.7)	49 (6.7)	0 (–)	49 (5.2)	0.001
Work overload	No data	No data	No data	220 (26.9)	63 (25.5)	283 (26.5)	0.36
Dry throat	13 (35.1)	3 (16.7)	16 (29.1)	210 (25.5)	26 (11.0)	236 (22.3)	0.001
Hoarseness	13 (36.1)	1 (5.9)	14 (26.4)	181 (21.2)	15 (5.6)	196 (17.4)	0.001
Pets at home	21 (56.8)	6 (33.3)	27 (49.1)	357 (43.3)	95 (38.1)	452 (42.1)	0.01
Indoor air problems at home	4 (14.8)	4 (22.2)	8 (17.8)	28 (4.9)	19 (8.6)	47 (6.0)	0.054

Crude rates, *n* (%) of hoarseness and background variables

### Phase 3 (Hospital study)

In the hospital with a severe IEQ problem, the prevalence of hoarseness was very high, 39.1%. In the reference hospital with a milder IEQ problem, the prevalence of hoarseness was significantly lower (23.3%), but still high, being at the same level as in the PHC units with an IEQ problem or in the pilot study exposure group. At the same time, however, the work overload/work-related stress levels were low in both hospitals, even lower than it was in the PHC units with or without an IEQ problem (Tables 2 and 3).

**Table 3:** Description of the hospital study (Phase 3)

	Exp. hospital	Ref. hospital	*p* value
No. of buildings	1	1	
*N*	973	226	
Mean age	42.73	42.65	
Men Women Total	72 (7.4) 901 (92.6) 973	24 (10.6) 202 (85.0) 226	0.074
Smokers	54 (5.5)	30 (13.2)	0.001
Passive smoking	29 (2.9)	11 (4.9)	0.15
Work overload	202 (20.8)	47 (21.1)	0.54
Work-related stress	121 (12.5)	33 (14.5)	0.27
Dry throat	354 (37.8)	49 (22.7)	0.001
Hoarseness	363 (39.1)	50 (23.3)	0.001
Pets at home	356 (36.3)	95 (41.9)	0.12
Indoor air problems at home	71 (6.6)	11 (7.9)	0.90

Crude rates (*n*, %) of hoarseness and background variables

However, the prevalence of work-related stress was much lower than the reported excessive workload in the material from Phase 2 (Table 2). The reported work-related stress and excessive workload were highly correlated in the hospital material (*p* < 0.001, data not shown). In the Phase 3 hospital study, the prevalence of hoarseness did not correlate with work-related stress (*p* = 0.94), shift work (*p* = 0.09), smoking (*p* = 0.79) or pet owning (*p* = 0.72).

### Phase 4—follow-up

Before the follow-up, four buildings with IEQ problems underwent thorough renovations: moisture damage was repaired, constructions with mold infestation were renewed, ventilation ducts cleaned, and a thorough cleaning of all surfaces was completed after the repair work. Subsequently, the prevalence of hoarseness diminished from 16.2 to 11.4%. The prevalence of hoarseness compared to the reference group was statistically significant prior to the repair. After the repair, the prevalence of hoarseness was somewhat higher than in the reference group, but the difference was no longer significant. The prevalence rate of a dry or sore throat was not altered and the difference with the reference group remained significant (Table 4).

**Table 4:** Description of the follow-up study of the PHC units before (1) and after the remediation (2) in Phase 4

	Exp. PHC units before repair (1)	PHC units reference group (1)	*p* value	Exp. PHC units after repair (2)	PHC units ref.group follow-up survey (2)	*p* value
No. of buildings	4	4		4	1	
*N*	275	272		328	102	
Mean age	44.3	44.4		44.2	44.2	
Men Women Total	26 (9.5) 249 (90.5) 275	16 (5.9) 256 (94.1) 272	0.12	44 (13.4) 284 (86.6) 328	8 (7.9) 93 (92.1) 102	0.13
Smokers	23 (13.4)	26 (10.5)	0.73	31 (10.9)	7 (7.2)	0.27
Passive smoking	26 (9.8)	9 (3.7)	< 0.01	16 (6.0)	1 (1.1)	0.13
Work overload	66 (24.1)	63 (25.5)	0.17	65 (22.7)	16 (16.5)	0.76
Sore throat	64 (24.1)	26 (11.0)	< 0.001	62 (22.6)	8 (9.0)	0.01
Hoarseness	44 (16.2)	15 (5.6)	< 0.001	38 (11.4)	5 (4.9)	0.09
Pets at home	114 (41.5)	94 (38.1)	0.28	117 (40.8)	32 (33.0)	0.32

Crude rates (*n*, %) of hoarseness and background variables. Surveys in the exposed groups and reference groups were performed in the same year in the PHC units with the IEQ problem and the unit with no IEQ problem and no intervention. The time between survey 1 and survey 2 was 2–3 years

### Time trend in the prevalence of hoarseness

In the pilot study, the prevalence of hoarseness in a non-damaged reference ward was 5.9% and in the exposed group 36.1%. In the larger study of PHC units, the respective values were 5.6% and 21.2%. Throughout this study, the prevalence of hoarseness has remained on the same low level in buildings with no IEQ problems (5.9% at the beginning of the study and 4.9% at the end of the study). In the PHC units with IEQ problems, the prevalence of hoarseness was 4–6 times higher. During the follow-up of PHC units after a thorough remediation, the crude prevalence of hoarseness decreased from 16.2 to 11.4%. One year after the remediation, the symptom level had still not reached the low level found in the buildings with no IEQ problem. At the end of the follow-up of the PHC units, the hoarseness experienced by employees in remediated buildings was 11.4% and in the reference group 4.9% (Tables 2, 3 and 4).

### The perceived IEQ factors

In Phases 1 and 2, in IEQ problem buildings, the employees reported significantly more often than the reference groups stuffiness, insufficient ventilation, unpleasant smells, dry air and dust/dirt on surfaces. In addition, visible signs of moisture damage were common (41.3%) in the IEQ problem buildings. Only noise and low temperatures occurred equally often in both groups (Table 5). Noise, dust, draught, varying temperatures and dry air correlated significantly with the prevalence of hoarseness.

**Table 5:** Subjective annoyance due to IEQ factors, Phase 1, 2 and 3

	Pilot study			PHC units				Hospitals		
	Exp.	Ref.	*p* value	Exp.	Ref.	Total	*P* value	Exp.	Ref.	*P* value
Draught	14 (40.0)	8 (47.1)	0.78	232 (30.4)	58 (25.0)	290 (29.1)	0.01	601 (65.3)	116 (55.3)	0.001
Too high temperature	14 (41.2)	3 (20.0)	0.27	153 (20.3)	36 (15.4)	189 (19.1)	0.001	358 (40.4)	62 (31.0)	0.01
Varying temperatures	14 (42.4)	4 (23.5)	0.24	159 (20.6)	37 (15.9)	196 (19.5)	0.001	465 (53.7)	85 (42.2)	0.03
Too low temperatures	8 (23.5)	2 (12.5)	0.61	92 (12.6)	27 (11.7)	119 (12.4)	0.42	354 (41.0)	71 (32.0)	0.41
Dry air	20 (57.1)	12 (70.6)	0.19	319 (41.2)	73 (30.9)	392 (38.8)	0.001	791 (84.0)	160 (76.5)	0.001
Insufficient ventilation	28 (75.7)	9 (50.0)		462 (77.5)	128 (52.5)	590 (70.2)	0.001	700 (76.6)	102 (49.3)	0.001
Stuffed indoor air	26 (70.3)	9 (60.0)	0.09	414 (51.3)	77 (32.5)	491 (47.0)	0.001	719 (77.4)	115 (54.1)	0.001
Unpleasant smells	18 (52.9)	9 (60.0)	0.87	265 (34.8)	65 (28.1)	330 (33.2)	0.001	362 (41.3)	64 (31.5)	0.01
Mold odor	19 (34.5)	4 (7.3)		70 (12.1)	8 (3.7)	78 (9.8)	0.001	330 (37.4)	32 (15.7)	0.001
Noise	6 (18.8)	0	0.20	150 (20.5)	48 (21.1)	198 (20.6)	0.20	462 (57.8)	108 (53.2)	0.18
Poor light, reflections	14 (41.2)	0	0.003	125 (17.1)	16 (7.2)	141 (14.8)	0.001	312 (35.6)	77 (38.1)	0.23
Dust, visible dirt	22 (61.1)	2 (13.3)	0.005	103 (14.2)	22 (9.7)	125 (13.2)	0.01	315 (35.8)	53 (25.7)	0.01
Visible moisture spots, current	4 (10.8)	3 (16.7)	0.41	282 (41.3)	43 (17.4)	325 (35.1)	0.001	408 (44.7)	42 (20.0)	0.001

Crude prevalence rates before the remediation for the exposure and reference groups (Phase 1 and 2)

In hospital buildings (Phase 3), draught, high or varying temperatures, dry air, stuffy air, insufficient ventilation, microbial odors and unpleasant smells were reported significantly more often in the exposure group than in the reference buildings. The differences were also significant as regards the occurrence of dusts and moisture spots. Perceived IEQ problems observed by employees are presented in Table 5.

### The effect of the remediations

In addition to hoarseness and throat symptoms, many other symptoms as well as asthma and allergic rhinitis diagnosed by a physician were investigated during the observational period 2007–18. Several symptoms of the respiratory tract, eyes and skin were significantly more prevalent in the IEQ problem buildings than in the reference groups. The difference was similar but not as large in the hospital study as in the PHC study (Table 6). Asthma and allergic rhinitis were on rather similar levels when those exposed were compared to those not exposed in Phases 1, 2 and 3.

**Table 6:** The prevalence of symptoms and asthma and allergic rhinitis (*diagnosed by a physician), Phase 1, 2 and 3

	Pilot study		PHC units				Hospital		
	**Exp.**	**Ref.**	**Exp.**	**Ref.**	**Total**	***p* value**	**Exp.**	**Ref**	***p* value**
Blocked nose, rhinitis	24 (64.9)	7 (38.9)	335 (40.6)	53 (21.8)	388 (36.3)	0.001	633 (66.3)	119 (54.0)	0.001
Cough	15 (41.7)	0	174 (21.5)	24 (10.2)	198 (19.0)	0.001	342 (36.5)	57 (26.0)	0.01
Dyspnea	5 (14.7)	0	53 (6.8)	4 (1.7)	57 (5.6)	0.01	96 (10.4)	16 (7.5)	0.27
Wheezing	1 (3.0)	0	32 (4.1)	2 (0.9)	34 (3.4)	0.03	56 (6.1)	1 (0.5)	0.01
Eye irritation	12 (33.3)	3 (17.6)	338 (40.9)	31 (13.3)	369 (34.8)	0.001	643 (67.1)	128 (57.9)	0.001
Facial skin irritation	14 (38.9)	3 (17.6)	258 (31.1)	42 (16.3)	300 (29.0)	0.001	556 (57.9)	96 (43.5)	0.001
Skin irritation in hands	10 (28.6)	8 (44.4)	319 (39.4)	69 (29.1)	388 (37.1)	0.001	650 (67.2)	119 (53.8)	0.001
Asthma*	4 (10.8)	2 (22.2)	76 (9.8)	19 (7.9)	95 (9.4)	0.36	68 (6.9)	19 (8.4)	0.45
Allergic rhinitis*	11 (29.7)	7 (43.8)	232 (29.8)	83 (34.3)	315 (30.9)	0.18	145 (14.8)	32 (14.1)	0.79

Crude prevalence rates in the exposure and reference groups, *n* (%)

In the Phase 4 follow-up study, the prevalence of symptoms in the IEQ problem buildings were clearly and significantly on higher level compared to the reference groups, with the exception of wheezing which was rare in both groups. After the remediation, the prevalence of respiratory symptoms was largely unchanged in the exposure groups. However, eye symptoms, and symptoms experienced on facial skin had diminished in the exposure group. Despite this, the differences between the exposure and reference group remained statistically significant before and after the repair with the exception of wheezing and dyspnea, which were quite rare (Table 7).

**Table 7:** The prevalence of symptoms (daily or every week), asthma and allergic diseases before the repair (1) and after the repair (2) in Phase 4

	Exp. PHC units before repair (1)	PHC units reference group (1)	*p* value	Exp. PHC units after repair (2)	PHC units ref.group follow-up survey (2)	*p* value
Blocked nose, rhinitis	108 (39.7)	53 (21.8)	0.001	108 (38.0)	20 (22.0)	0.01
Cough	53 (20.0)	24 (10.2)	0.02	50 (18.0)	11 (12.4)	0.06
dyspnea	9 (3.5)	4 (1.7)	0.57	8 (2.9)	1 (1.1)	0.81
Wheezing	3 (1.1)	2 (0.9)	0.99	3 (1.1)	0 -	0.69
Eye irritation	77 (28.8)	31 (13.3)	0.001	67 (23.7)	8 (8.8)	0.001
Facial skin irritation	78 (30.2)	42 (17.7)	0.01	73 (25.9)	11 (11.3)	0.01
Skin irritation in hands	110 (41.4)	69 (29.1)	0.01	119 (42.0)	24 (24.7)	0.02
Asthma	21 (7.7)	19 (7.9)	0.54	24 (9.0)	8 (8.8)	0.58
Allergic rhinitis	92 (33.9)	83 (34.3)	0.93	108 (40.3)	35 (38.9)	0.55

The effect of remediation in the PHC units with IEQ problem compared to reference PHC units. Crude rates (*n*, %), statistical Chi-Square (*Χ*^2^) Test

In addition, the prevalence of asthma was at the same level among the exposed and the reference group prior to the renovation. The prevalence of asthma did not change during the follow-up and the difference between the IEQ problem group, and the non-problem group was not significant in pre-and post-repair surveys. The same was true for the prevalence of allergic rhinitis (Table 7).

We found that hoarseness and work-related stress were not correlated in the years between 2008 and 18 when Phase 2 of the study was conducted in the PHC units. Those employees working in IEQ-problem buildings had a higher prevalence of hoarseness compared to the reference group, even though the work-related stress was quite high in both groups (Table 2).

In 2011 in Phase 3, the prevalence of hoarseness was very high in the hospital with a severe IEQ problem, however, in the reference hospital with a milder IEQ problem, the prevalence of hoarseness was at the same level as in the PHC units with IEQ problem. At the same time, the stress level was low in both hospitals, even lower than in the PHC units with or without an IEQ problem. In phase 4, after the renovations, the hoarseness had decreased, but it was still higher than in the reference units, even though the workload in both remained at the same level.

The workload after the renovation was 22.7% and 16.5%, respectively. The prevalence of hoarseness did not correlate with work-related stress in the hospitals. Work-related stress was at a higher level in the PHC units than in the hospital with a higher level of hoarseness.

### Risk factors for hoarseness

In a logistic regression model of the follow-up data from the PHC units with and without IEQ problems, age, smoking, allergic rhinitis, asthma and unrepaired IEQ problems in the workplace were statistically significant factors for hoarseness. Women had a higher risk for hoarseness, but the difference was not significant. The risk of hoarseness decreased with increasing age. Smoking increased the risk of hoarseness. The association between asthma and hoarseness was strong and highly significant, but the confidence interval was wide. Remediation of the building reduced the risk of hoarseness, and unrepaired IEQ problems were a statistically significant risk factor for hoarseness, when age, gender, allergic rhinitis, asthma, smoking, and pets were adjusted in the logistic regression model (Table 8)

**Table 8:** The risk for hoarseness in PHC units associated with the remediation of the buildings in the follow-up study

	*N*	OR	95% CI interval	*p* value
Age	757	0.942	0.915–0.970	0.001
Gender, female Male		1.665 1	0.619–4.480	0.31
Pets No pets		0.591 1	0.343–1.019	0.06
Smoking No smoking		1.659 1	1.229–2.239	0.001
Excessive workload Less workload		0.88 1	0.614–1.262	0.488
Allergic rhinitis No all.rhinitis		1.700 1	1.013–2.853	0.044
Asthma No asthma		8.465 1	4.417–16.226	< 0.001
Unrepaired IEQ problem Repaired or no damage		1.877 1	1.361–2.588	< 0.001

OR and 95% confidence intervals in a logistic regression model adjusted for age, gender, allergic rhinitis, asthma, excessive workload, smoking and having pets

## Discussion

To the best of our knowledge, no similar studies on the time trends or long-term follow-up studies of whole workplaces have been published previously about the prevalence of hoarseness and risk factors for vocal problems among health care professionals. Nevertheless, from a problem-solving perspective, long-term follow-ups are extremely important, nearly essential. However, follow-up research is laborious and time-consuming and has its limitations for instance dropouts over the time. Because the staff at workplaces is always changing, some retire, some change jobs, etc., this study was conducted as an open cohort study where the entire work force A in time point 1 were compared to respective group B in time point 2. These materials are considered as independent samples. Additionally, in each follow-up time point, a paired sample analysis was performed always when the respondent could be identified by name. The reported health status of the person N.N. was compared to respective person in the follow-up at time point 2 and 3. The statistical analysis of paired observations gives stronger statistical power than comparison of the larger study material at time points 1 and 2. Of course, as time goes by, the number of individuals participating all surveys is getting smaller and statistical significance is more unlikely to be observed. We were also interested of the health of ‘new’ workers entering the cohort, how they would consider their health status in the remediated building. By following solely the original population at time point 1, would possibly lead to bias in the results due to so-called ‘healthy worker effect’.

Another limitation here was that the PHC units were quite small, having only 20–150 employees per building. Large hospitals often have thousands of employees so the statistical power in hospital studies is much stronger. Also, the fact that the respondents were aware of the exposure can be considered as a limitation. However, but this was impossible to avoid, because in most cases the employees could see the damage in the structures and/or experience the unpleasant odors. They were aware of the remediation especially if they were transferred to other premises or if they could see and hear the demolition and construction work in the building and were exposed to dust, fibers and noise.

Moreover, the small number of reference buildings compared to the number of buildings with IEQ problems is also a clear limitation. However, buildings with no problems are not easy to find, and it is especially difficult to motivate the building owners to do costly examinations, such as analyze microbial samples and chemicals in the indoor environment. In our material, only one reference building was motivated to participate in the repeated surveys, probably because other employers and employees could not see any immediate benefit from participation as a reference group.

In addition to limitations, this study also has its benefits. One of the major benefits was the high or very high participation rate in almost all surveys. The participating units were very committed and cooperative during the field studies and participated well in the clinical investigations. The results of the clinical studies have been analyzed and will be published in a separate article. Another benefit is the longitudinal study setting instead of a cross-sectional study as is the case with many of the previous investigations. Another beneficial factor is also that the participating PHC units were in different parts of the country, not only in one town or small geographic area. Based on our previous studies we consider this material representative of the middle and south of Finland. Our results are in line with our studies on a large nation-wide random sample of health care professionals, the results of which have already been published (Vilén et al. 2022; Vilén and Putus 2021). The current study was also supported by a multidisciplinary study group including building professionals thus ensuring a reliable classification of the participating PHC units and hospitals into two groups: those buildings with IEQ problems and healthy buildings. These professionals in construction engineering also controlled and supervised the remediation work in the PHC units.

However, in general it can be stated that hoarseness is a common symptom in adults, nearly one in three adults suffer from it at some point in their lives. The point-prevalence is estimated to be somewhere between 6.6 and 10% (Cohen et al. 2012; House and Fisher 2017; Martins et al. 2016; Pylypowich and Duff 2016; Roy et al. 2005), which is even a little higher than our results in buildings with no IEQ problems. However, unfortunately, based on our findings the prevalence of hoarseness is 3–6 times higher in PHC units with indoor air problem. Our study shows that the prevalence of hoarseness has remained the same for approximately 10 years in buildings with good IEQ and buildings with no significant moisture or microbial damage.

Based on our findings, the statistically significant risk factors for hoarseness were age, smoking, allergic rhinitis, asthma and unrepaired IEQ problems in the workplace. It is well-known that smoking irritates and dries vocal cord mucosa which can result in an abnormal voice. In addition, the finding that asthma and allergic rhinitis are risk factors for hoarseness are in line with many previous studies. The role of asthma medication cannot be distinguished from the effect of asthma, but hoarseness is a common side-effect of asthma medication, especially corticosteroids. Moreover, antihistamines used for allergies have a mucosal drying effect, which may also increase the risk of hoarseness (House and Fisher 2017). However, the prevalence of asthma and allergic rhinitis did not alter during the follow-up, and they were approximately at the same level in all units.

The remediation of buildings has a clear and statistically significant beneficial effect on the prevalence of hoarseness, as well as on perceived IEQ problems. The prevalence of hoarseness after the remediation was lower than before remediation, although still at a somewhat higher level compared to reference units with no known exposure. The reported stuffiness, unpleasant smells, too low temperature, and signs of moisture damage diminished. This finding is in line with previous findings in the remediation of school buildings and its effect on teachers’ health (Patovirta et al. 2004; Putus et al. 2021).

One study of teachers, Vertanen-Greis et al. (2018) found an association with vocal problems and work-related stress. However, we did not find any clear connections between hoarseness and work overload or in the hospital study with work-related stress. In addition, in our own follow-up study of teachers a decline in hoarseness and an increase in work-related stress was observed after the remediation of school buildings (Putus et al. 2021). In our current material, the workload in PHC units was higher than in hospitals. We found no previous comparative study on workload or stress in different workplaces in the health care sector. Based on this study, the type of work (hospital vs. PCH) correlated more clearly with the level of stress than with IEQ problems.

## Conclusion

Over 10 years of the follow-up it was observed that the prevalence of hoarseness had not increased in workplaces with good IEQ, it has remained between 4.9 and 5.6% throughout the follow-up period. However, the prevalence of hoarseness was found to be 3–6 times higher in buildings with indoor air problems. Based on this study, the risk factors for hoarseness were age, smoking, allergic rhinitis, asthma and unrepaired IEQ problems in the workplace. The renovation of the building reduced the risk of hoarseness and the reporting of IEQ problems, although the levels of both were still slightly higher than in reference buildings.

## Data Availability

Due to privacy and ethical concerns, supporting data cannot be made openly available.
